# Effect of positive feedback from patients on resident doctor's well-being

**DOI:** 10.6026/973206300215055

**Published:** 2025-12-15

**Authors:** Priyanku Saikia

**Affiliations:** 1 Department of Accident and Emergency, TIMeS Hospital, Tezpur, Assam, India

**Keywords:** Patient gratitude, resident doctors, burnout, emotional exhaustion, depersonalization, personal accomplishment, well-being, positive feedback, resilience, job satisfaction

## Abstract

The effect of patient's gratitude on the well-being of Residents Doctors, especially in helping to reduce burnout is of interest. Therefore, it is of interest to assess the effect of gratitude on emotional exhaustion, depersonalization and a sense of personal accomplishment among trainees using a mixed-methods approach. We show that gratitude can lower emotional exhaustion and strengthen feelings of accomplishment, although its effect on depersonalization was not statistically significant. Thus, positive patient interaction and gratitude based practices help doctor's mental well-being.

## Background:

Burnout has become a major concern within healthcare systems globally, with resident doctors being particularly affected due to prolonged working hours, high patient loads, emotional strain and limited recovery time [1, 5]. Burnout is characterised by emotional exhaustion, depersonalisation and a reduced sense of personal accomplishment, all of which adversely affect doctors' mental health, professionalism and the quality and safety of patient care [5]. High burnout levels among residents have been linked to reduced job satisfaction, impaired doctor-patient relationships and increased risk of clinical errors [1, 2]. Although several strategies have been introduced to address burnout-such as workload regulation, stress-management programmes and institutional well-being initiatives-their overall impact has been modest and often insufficient, particularly among doctors in training [5, 6]. Persistent burnout despite these interventions suggests the need for additional approaches that address the emotional and psychological dimensions of clinical work rather than focusing solely on structural changes [6, 10]. Recent evidence indicates that psychosocial factors, including positive reinforcement and supportive workplace environments, play an important role in healthcare professionals' well-being [7]. Patient expressions of gratitude represent a meaningful form of positive feedback that acknowledges clinicians' efforts and reinforces the value of their professional contribution [12]. Such interactions have been associated with improved motivation, emotional resilience and job satisfaction among healthcare workers [8]. Positive patient feedback has also been shown to support empathy and strengthen professional fulfilment, particularly in demanding clinical settings [11]. Emerging research highlights that cultivating positive workplace cultures and reinforcing meaningful aspects of clinical practice can contribute to improved physician well-being and resilience [14]. Approaches that focus on emotional validation, professional recognition and positive interactions during medical training may help mitigate certain components of burnout, especially emotional exhaustion and reduced personal accomplishment [13]. However, despite growing interest in these areas, there remains limited empirical evidence specifically examining the impact of patient-derived positive feedback on burnout outcomes among resident doctors. Most existing studies focus on burnout prevalence, workload factors, or general well-being measures, with relatively little attention given to patient gratitude as a potential protective factor [4]. Understanding how positive feedback from patients influences resident doctors' emotional health, job satisfaction and professional engagement may help identify practical, low-cost interventions that complement existing burnout reduction strategies and support sustainable well-being within clinical training environments [8, 10]. Therefore, it is of interest to effect of positive feedback from patients on resident doctor's well-being. Figure 1 (see PDF) outlines the overall study design, showing how resident doctors were assessed for burnout before and after exposure to patient expressions of gratitude, using a mixed-methods approach that combines quantitative burnout scores with qualitative interviews. This figure helps clarify the step-by-step flow of the methodology used in the study.

## Methodology:

A mixed-methods study design was used to examine the relationship between patient expressions of gratitude and burnout among resident doctors, combining quantitative burnout assessment with qualitative exploration of lived experiences from pre-collected datas.

## Participant selection:

Resident doctors working across multiple clinical specialties were recruited for the study. A total of 100 residents with at least six months of continuous clinical experience were included to ensure adequate exposure to patient interactions. Participation was voluntary and residents with prolonged absence during the study period or ongoing treatment for burnout-related conditions were excluded to minimise confounding influences.

## Data collection:

## Quantitative data collection:

Burnout was assessed using the Maslach Burnout Inventory, focusing on emotional exhaustion, depersonalisation and personal accomplishment. Assessments were conducted at baseline and repeated after a six-month interval. Participants documented instances of patient gratitude encountered during routine clinical work. The frequency of gratitude was categorised using a structured scale, allowing comparison between the extent of patient appreciation and changes in burnout scores over time.

Research will examine this relationship between patient gratitude frequency and MBI scores using the following formula:

(see PDF)

## Results:

The research study on resident doctors' burnout and well-being levels in relation to patient gratitude has uncovered essential findings. The research results establish a noticeable association where patient gratitude frequency associates with decreased burnout in residents through their experience of positive feedback which both combats emotional exhaustion and enhances job satisfaction [9]. The research shows that patients expressing gratitude directly correspond to lower results in physician burnout surveys. Doctors who experienced frequent gratitude from patients and their families demonstrated substantial decreases in their emotional exhaustion and depersonalization which make up burnout symptoms [8]. Medical staff who received expressions of gratitude indicated a stronger bond with their patients and stronger work-related meaning. Medical staff members who receive no or minimal patient gratitude developed more severe burnout symptoms leading to disinterest in work duties and disconnect from patient. Doctors who received appreciation from their patients scored higher when measuring emotional empathy as well as compassion and their professional fulfillment and job satisfaction ratings. The positive feedback served two purposes: it prevented burnout and drove doctors to enhance their patient relationships which subsequently enhanced care quality. A summary of the relationship between patient expression of gratitude and burnout symptoms appears in Table 5 (see PDF). Three data groups exist according to the number of patients expressed gratitude in this study designated as low moderate and high. Lifestyle assessment scores reflect how doctors experience burnout and job satisfaction depending on whether they received little or much gratitude from patients.

As shown in Table 7 (see PDF), residents who frequently received gratitude from their patients reported noticeably lower burnout. The same group also experienced higher job satisfaction and stronger empathy scores. In contrast, those who rarely received gratitude showed higher burnout and felt less fulfilled in their role. Overall, the pattern in Table 7 (see PDF) highlights how meaningful appreciation from patients can positively influence well-being and emotional connection in medical practice. Frequent patient expressions of gratitude resulted in doctors reporting low burnout together with high levels of satisfaction and displayed empathy toward patients. Medical professionals who received gratitude from their patients relatively seldom or never showed higher rates of burnout alongside decreased satisfaction scores. Healthcare provider psychological wellness together with professional capability profits significantly from positive feedback according to these results. The research shows that patient gratitude works as a beneficial method to decrease work-related stress and enhance resilience among resident doctors although it does not function as a complete solution for burnout. The research shows that healthcare institutions might enhance medical practitioner wellness together with patient results through cultivating appreciation throughout their professional environment.

## Discussion:

This study explored the association between patient expressions of gratitude and burnout-related outcomes among resident doctors. The findings suggest that positive feedback from patients is linked to lower burnout levels, particularly through reduced emotional exhaustion and improved professional well-being. These results support existing evidence that psychosocial factors play an important role in sustaining doctors' mental health during training [1, 5]. Emotional exhaustion emerged as the most affected burnout dimension, with residents reporting lower exhaustion when they experienced frequent patient appreciation. This finding is consistent with previous research indicating that positive reinforcement and acknowledgment can help buffer the emotional demands of clinical work [3, 9]. While patient gratitude cannot replace systemic interventions, it may provide meaningful emotional support within high-pressure environments. The study also demonstrated a positive association between patient gratitude and personal accomplishment. Residents who felt appreciated reported greater professional fulfilment and motivation, suggesting that positive patient interactions may reinforce a sense of purpose during training [7, 11]. In contrast, the effect on depersonalisation was less pronounced, indicating that this dimension may be more strongly influenced by workload and organisational factors rather than individual positive experiences alone [5, 10]. Patient gratitude was additionally associated with improved job satisfaction and morale. Residents described greater engagement with their work and more positive doctor-patient relationships when appreciation was expressed. These findings align with earlier studies linking positive feedback to enhanced job satisfaction and emotional resilience among healthcare professionals [4, 12]. Several limitations should be considered when interpreting these findings. The observational design limits causal inference and self-reported measures may introduce response bias [3]. In addition, the frequency of gratitude was measured without distinguishing between different forms of appreciation, which may vary in psychological impact. The sample size and single-setting design may also limit generalizability [9, 13]. Despite these limitations, the study highlights patient gratitude as a simple, low-cost factor that may complement existing burnout reduction strategies. Encouraging positive patient feedback alongside organisational support may contribute to more sustainable approaches to improving resident doctors' well-being and professional satisfaction [10, 14].

## Future scope:

The research findings create multiple opportunities for academic investigations and clinical applications in healthcare facilities. Structured patient appreciation programs represent a promising approach for developing systems which promote gratitude among medical patients. Medical institutions and hospitals should implement "thank you" program initiatives to let patients show appreciation personally to their care providers so doctors receive improved emotional support [9]. Further inquiry should investigate how much time patient expressions of gratitude endure to influence doctor burnout. A sustained assessment of burnout and job satisfaction and well-being through time would help determine whether doctors maintain high mental health benefits due to gratitude exposure throughout the observation period. Research needs to determine which gratitude methods between verbal expressions and written notes and gestures yield the strongest effects for doctor well-being. Attention should be devoted to establishing how patient gratitude influences different healthcare specialties. The analysis investigated resident doctors, yet different medical specialties show different levels of patient thankfulness contact so their responses to gratitude might vary by specialization work. Medical personnel working in emergency or critical care settings probably have different reactions to patient gratefulness than professionals from more standard hospital wards. A possible exploration exists for implementing patient gratitude into training curricula for healthcare service providers. Educating resident physicians about understanding and appreciating patient gratitude allows them to efficiently receive positive feedback thus strengthening their professional endurance and decreasing practice-related stress.

## Conclusion:

Patient expressions of gratitude improve emotional exhaustion in resident doctors while simultaneously boosting their sense of personal accomplishment. Gratitude stands as an essential instrument for professionals in high-stress fields to enhance their well-being, but it does not resolve all burnout elements. Medical organizations can easily implement patient gratitude as an effective method to maintain their healthcare staff's mental health. Researchers should sustain their efforts to examine gratitude's effectiveness in decreasing burnout symptoms and building healthcare provider wellness in clinical environments.
